# S4HARA: System for HIV/AIDS resource allocation

**DOI:** 10.1186/1478-7547-6-7

**Published:** 2008-03-26

**Authors:** Arielle Lasry, Michael W Carter, Gregory S Zaric

**Affiliations:** 1 Department of Mechanical and Industrial Engineering, University of Toronto, Toronto, ON, M5S 3G8, Canada; 2 Ivey School of Business, University of Western Ontario, London, ON, N6A 3K7, Canada

## Abstract

**Background:**

HIV/AIDS resource allocation decisions are influenced by political, social, ethical and other factors that are difficult to quantify. Consequently, quantitative models of HIV/AIDS resource allocation have had limited impact on actual spending decisions. We propose a decision-support System for HIV/AIDS Resource Allocation (S4HARA) that takes into consideration both principles of efficient resource allocation and the role of non-quantifiable influences on the decision-making process for resource allocation.

**Methods:**

S4HARA is a four-step spreadsheet-based model. The first step serves to identify the factors currently influencing HIV/AIDS allocation decisions. The second step consists of prioritizing HIV/AIDS interventions. The third step involves allocating the budget to the HIV/AIDS interventions using a rational approach. Decision-makers can select from several rational models of resource allocation depending on availability of data and level of complexity. The last step combines the results of the first and third steps to highlight the influencing factors that act as barriers or facilitators to the results suggested by the rational resource allocation approach. Actionable recommendations are then made to improve the allocation. We illustrate S4HARA in the context of a primary healthcare clinic in South Africa.

**Results:**

The clinic offers six types of HIV/AIDS interventions and spends US$750,000 annually on these programs. Current allocation decisions are influenced by donors, NGOs and the government as well as by ethical and religious factors. Without additional funding, an optimal allocation of the total budget suggests that the portion allotted to condom distribution be increased from 1% to 15% and the portion allotted to prevention and treatment of opportunistic infections be increased from 43% to 71%, while allocation to other interventions should decrease.

**Conclusion:**

Condom uptake at the clinic should be increased by changing the condom distribution policy from a pull system to a push system. NGOs and donors promoting antiretroviral programs at the clinic should be sensitized to the results of the model and urged to invest in wellness programs aimed at the prevention and treatment of opportunistic infections. S4HARA differentiates itself from other decision support tools by providing rational HIV/AIDS resource allocation capabilities as well as consideration of the realities facing authorities in their decision-making process.

## Background

Available funding for HIV and AIDS in low- and middle-income countries is estimated at US$27 billion in total for the years 2005 to 2007. However, that amount represents only 60% of the resource requirements estimated at US$45 billion in the same period [1]. Though funding has increased dramatically, the resource needs for an effective global response to the AIDS epidemic have increased even further.

### Rational approaches HIV/AIDS resource allocation

Resource allocation can be defined as the process of distributing funds or resources among intervention programs that are competing for the same budget. There are several types of rational models that can be used to support the decision-making process for HIV/AIDS resource allocation. Equity is perceived as an important value representing fairness and distributive justice [2-4]. Simple resource allocation models can be based on equity criteria such as an allocation proportional to the number of HIV/AIDS cases in different target groups [5,6]. Resource allocation models can be based on league tables which suggest allocating funds to interventions in ascending order of their cost-effectiveness ratios until the budget is exhausted [7].

More comprehensive approaches to resource allocation include simulation models used to project the epidemic over time and compare the outcome of alternative allocation scenarios [8,9]. A number of HIV/AIDS resource allocation models are formulated as an optimization problem [10-13]. The problem is usually stated as one of choosing the amount to be invested in several interventions to optimize total health benefits subject to a budget constraint.

Despite the increased acceptance of such rational models and their potential to produce very good results, their impact on health care resource allocation in practice has been rather limited [14-16]. In addition, there is evidence that actual spending decisions tend to deviate significantly from what rational models might suggest [17].

### Limitations of rational models for HIV/AIDS resource allocation

Several reasons have been discussed regarding the practical usability of rational resource allocation models. First, models are often complex and there is limited data or capacity to use them in low-income settings. In developing countries, barriers to the use of quantitative decision models in health care include a shortage of trained analysts and other personnel with the capacity to manipulate models, as well as a lack of awareness of models and their contributions [18,19]. Anderson and Garnett argue that emphasis on the elegance of the formulation and analysis of infectious disease models rather than on practical relevance, combined with inadequate knowledge in mathematics and statistics, creates a chasm between the researchers creating models and the decision-makers originally intended to use them [20]. In a study of the influence of mathematical modeling of HIV/AIDS on policies in the developing world, Stover concludes that policy makers often think that "modeling is not understandable, answers the wrong questions or suggests unrealistic solutions [21]."

Second, resource allocation decisions can not be made in a socio-political vacuum [22]. Resource allocation practices are subject to the influence of numerous social, ethical, political and other non-quantifiable considerations, yet rational models do not consider these influencing factors [23,24]. For example, there is a major policy debate on whether HIV funds are best spent on treatment or prevention. Marseille et al. argue that if the goal is to minimize total loss of life then the primary focus of HIV spending in sub-Saharan Africa should be prevention [25]. Creese et al. also make a case for prioritization of prevention over treatment based on a review of cost-effectiveness studies of HIV/AIDS interventions in Africa [26]. Both of these studies were criticized for not considering the social environment. Their critiques argue that the decision to treat cannot be based solely on the perspective of cost implications, but rather it should involve humanitarian considerations and a societal moral obligation to treat [27-29].

Third, rational models of resource allocation are useful to optimize the expected outcome for a single decision-making authority [30]. However, the decision-making process for HIV/AIDS resource allocation involves several decision-makers each with their own goals, priorities, processes and level of influence. These decision-makers include donors, advocacy groups, non-government organizations (NGOs), government agencies and local communities [24]. When many decision-makers are involved resource allocation models should be used as a means for structuring the problem so that conflicts can be handled constructively [30]. Baltussen and Niessen propose a multi-criteria decision making approach to guide healthcare resource allocation [31].

In a study of the use of operations research models in developing countries, Ravn and Vidal suggest that education in industrialized countries produces researchers who believe that to be scientific one ought to be politically neutral; but that both scientific analysis and political engagement are necessary to implement appropriate models in developing countries [32]. Despite the influence of qualitative criteria, policy models can provide important input to the public health policy making process, particularly when resources are scarce [33,34]. More priority setting tools are necessary, including those based on principles of cost-effectiveness, however, these tools should be attuned to decision-makers' needs, society's preferences and local circumstances [35].

In view of these limitations to the use and usability of rational resource allocation models, we propose a decision-support System for HIV/AIDS Resource Allocation (S4HARA) that takes into consideration both principles of efficient resource allocation and the role of non-quantifiable influences on the decision-making process for HIV/AIDS resource allocation. S4HARA enables decision-makers to select from several rational models of resource allocation depending on availability of data and level of complexity. We validate S4HARA by demonstrating its application in the context of a health care clinic in South Africa.

The remainder of this paper is organized as follows: we begin with a description of S4HARA; we then apply the system to a primary health care clinic in South Africa and describe the results. Finally, we conclude with some recommendations, known limitations and suggestions for future work.

## Methods

S4HARA is a four-step spreadsheet-based decision-support system for allocating funds to HIV/AIDS programs at a local level. S4HARA is aimed at local government agencies, non-governmental organizations or public health institutions currently offering HIV and AIDS programs in a low-income community. Though the primary user of S4HARA is the person in charge of budget planning within a given organization, consultation with several key contacts is required to gather input for the construction of a S4HARA model.

**Figure 1:**
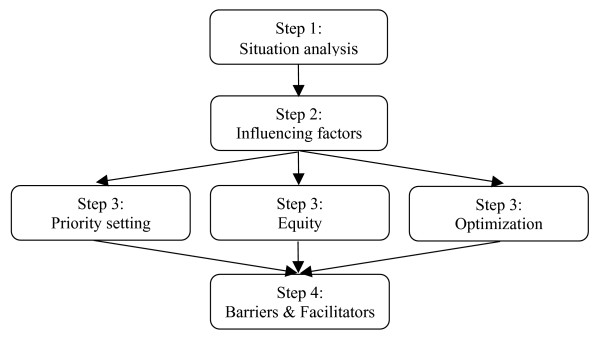
The 4 steps of S4HARA.

The flow diagram in Figure 1 illustrates the fours steps involved in S4HARA. The first step, situation analysis, consists of collecting data related to the target population and the HIV/AIDS programs offered. The second step serves to identify the factors that currently influence HIV/AIDS resource allocation decisions. The third step requires prioritizing the HIV/AIDS programs. The last steps combines the output of the second and third steps to create a comprehensive picture which highlights the influencing factors that act as barriers or facilitators to the results suggested by the rational resource allocation approach. This last step assists in the formulation of actionable recommendations intended to improve HIV/AIDS resource allocation.

S4HARA is designed to run on Microsoft Excel. Excel was selected as the platform for running S4HARA because of its widespread availability.

### Step 1: Situation analysis

In this step the user is required to collect data including population size, HIV prevalence, number of AIDS cases, the resource allocation planning horizon and the total budget. For each HIV/AIDS program offered by the organization, the user will compile the available data related to program utilization rates, such as the number of visits, material expenses and, if possible, program costs and outcome data. The WHO-CHOICE project is a useful source for identifying regional estimates of intervention costs and outcomes [36]. If cost-effectiveness analysis of the HIV/AIDS interventions is conducted, it should conform to recognized guidelines cost-effectiveness analysis [19,37,38]. The extent of the data collected will determine which of the resource allocation models could be used in the third step.

### Step 2: Influence diagram

Influence diagrams can be useful for illustrating problems that are difficult to quantify. In this step, an influence diagram is used to create a graphical representation of the factors that bear a positive or a negative influence on the implementation of the HIV/AIDS programs offered. The output of this step will be used as input to the final step.

#### Nodes

The influence diagram is composed of two types of nodes: program nodes representing the HIV/AIDS programs offered; and factor nodes representing the influencing factors. Program nodes are predefined based on the results of the situation analysis (Step 1).

The user is provided with a default set of nodes based on previous research. In previous research, we identified several bodies or groups that influence the decision-making process for HIV/AIDS resource allocation in low-income settings, including: donors, advocacy groups, NGOs, government agencies, communities and the media [24]. We also determined that many intangible factors influence the allocation of HIV/AIDS resources, for example, political power, relationships, leadership, ethics, culture and religion [24]. The user is given the option to customize or add factor nodes.

#### Creating the influence diagram

Nodes can be connected by two types of arcs; positive arcs are green and represent a positive influence between a factor node and a program node, and negative arcs are red and represent a negative influence between a factor node and a program node.

By default the influence diagram contains both program nodes and factor nodes but no arcs. For every combination of factor node and program node, the user must assess whether the factor bears an influence on the allocation of resources to the program and if so, whether it is a positive or a negative influence. Accordingly, the user will add positive and negatives arcs to the influence diagram. For example, the user may ask: "Does religion have an influence on the implementation of a condom distribution program? And, if so, is it a positive influence which encourages condom distribution or a negative influence which impedes condom distribution?" To improve the accuracy of answers, the user should consult with as many qualified respondents as possible. The thickness of the arcs should be used to determine the relative importance of the influence.

### Step 3: Rational model for resource allocation

In this step, the user selects and applies one of three HIV/AIDS resource allocation methods: priority setting, equity or optimization.

#### Priority setting

We define priority setting as an ordinal ranking of the programs according to the situation analysis. This approach does not require cost or outcome data and is recommended if information on current levels of funding or utilization for each program is limited.

In S4HARA, priority setting entails assigning a prescriptive priority level to each of the HIV/AIDS programs offered indicating what the priority level ought to be for each program. Each program is also assigned a current priority level relating the level of priority currently given to each program. The highest level is 1, 2 is the next highest level, etc. and each level should only be used once. The levels are ordinal ranks and do not express a quantitative measure. The difference between prescriptive and current priority levels of each program is used in the final step to determine if programs are over- or under-funded. Priority levels should be determined by a consensus among all qualified respondents. If consensus cannot be reached, priority setting can be mediated by vote or by seeking expert opinion.

Current priority levels should be set in view of the situation analysis and the user's perception of the target population needs, the programs' current clients and the program outcomes. Prescriptive priority levels can be elicited using the following two questions:

1) Hypothetically, if $10,000 were available, which of the HIV/AIDS programs offered would you put it towards? Rank the answer as 1 and consider the remaining programs only.

2) Hypothetically, if $10,000 were available, which of the remaining HIV/AIDS programs offered would you put it towards? Rank the answer as the next highest priority and consider the remaining programs only. Repeat this question until all HIV/AIDS programs are ranked.

If priority setting is chosen as the rational method of resource allocation, it is assumed that actual levels of spending for each program are unknown. Current priority levels should be based on information collected in the situation analysis including program utilization. Assigning current priority levels consists in ranking the HIV/AIDS programs offered relatively according to the current priority given to each program.

#### Equity

Several approaches can be used to define an equity-based heuristic allocation of HIV/AIDS resources. We define equity as an allocation proportional to the maximum allocation to each program within the planning horizon. The maximum allocation can be based on the absorptive capacity for an intervention, or, it can be calculated as the product of the unit cost of an HIV/AIDS program and the maximum number of people eligible for that program within the planning horizon. The information necessary to establish the maximum allocation is program-specific. For example, the maximum allocation to ART depends on the number of AIDS cases in the population and the annual cost of an ART regimen, while the maximum allocation to VCT depends on the adult population targeted and the unit cost of VCT. The equity allocation consists of partitioning the total budget proportionally according to the maximum allocation to each program. The difference between the equity allocation and the current allocation to each program is the main output used in the final step.

An equity-based heuristic allocation of HIV/AIDS resources is recommended if the organization values the notion of fairness associated with equity above the notion of efficiency associated with optimization, or if cost-effectiveness data for the programs considered are not available.

#### Optimization

This approach to resource allocation requires all data outlined in the situation analysis and uses cost-effectiveness ratios to maximize health outcomes. Cost and outcome data for the HIV/AIDS programs are used to determine the cost-effectiveness ratios. However, if cost or outcome data are not available, then cost-effectiveness ratios may be identified through literature searches. The denominator of the cost-effectiveness ratios should be expressed in disability-adjusted life years (DALYs) or some other common measure of outcome.

The optimization problem is then formulated as a linear program where the objective function aims to maximize the total number of DALYs gained, subject to a total budget constraint. The decision variables are the amounts to invest in each of the HIV/AIDS programs. There may also be maximum and minimum funding levels for each program. This allocation problem is equivalent to a knapsack problem and is easily solved by selecting programs in order of their cost-effectiveness ratios until the budget is exhausted [39]. Sensitivity analysis should be performed by varying the cost-effectiveness ratios and the budget constraints to evaluate the robustness of the results obtained. A description of the mathematical optimization model is provided in the Appendix.

### Step 4: Barriers and Facilitators

In this last step, the background color of program nodes in the influence diagram drawn in Step 2 are used to highlight the output of Step 3. The background color of program nodes are set to bright green, light green, light red and bright red depending on whether a program should receive significantly more, slightly more, slightly less or significantly less funding, funding than the current allocation.

Program nodes with matching coloured incoming arcs indicate that the influencing factors are a facilitator to the resource allocation objective while program nodes with mismatched coloured incoming arcs indicate that influencing factors act as a barrier to the objective set by the resource allocation model.

Recommendations for improving the allocation of resources to HIV/AIDS programs are not automated. Rather, S4HARA assists in the formulation of actionable recommendations intended to improve HIV/AIDS resource allocation by creating a comprehensive picture highlighting the influencing factors that work either towards or against the solution derived in Step 3. The user examines the diagram in Step 4, detects the barriers and facilitators and identifies their source. The way these sources act as an impediment or an endorsement to an improved allocation must be interpreted. The user can then formulate actionable recommendations to alleviate the barriers and use the facilitators to encourage the allocation of resources.

## The case of the kwaDukuza primary health care clinic

We illustrate the use of S4HARA with an example based on the kwaDukuza Primary Health Care (PHC) clinic. The municipality of kwaDukuza has a population of 170,000 and is located on the Indian Ocean coastline in the south-east of South Africa, near Durban.

As part of a previous case study, we conducted 35 key informant interviews and collected documents relevant to HIV/AIDS programs and budgets in kwaDukuza over a six-week period during March and April, 2005 [24]. Interview candidates represented national, provincial and local government institutions as well as NGOs, advocacy groups and academia. Interviews consisted of ten open-ended questions addressing resource allocation issues allowing candidates to speak openly about the realities and complexities of the HIV/AIDS resource allocation process in kwaDukuza.

In 2004, the antenatal HIV prevalence rate in kwaDukuza was estimated at 40% [40]. In the municipality of kwaDukuza, HIV and AIDS programs are delivered through health care clinics, a hospital and non-governmental organizations. Our example focuses on the kwaDukuza PHC clinic, the largest clinic in the municipality.

### Step 1: Situation analysis

Demographic information on the municipality of kwaDukuza and data on the clinic and its HIV/AIDS programs were collected [24]. The kwaDukuza PHC clinic serves approximately 61,000 people in kwaDukuza. To estimate this figure, we assess the proportion of visits made to the kwaDukuza PHC clinic relative to the number of visits made to all clinics in the municipality and then multiply by the population of kwaDukuza. During 2004, a total of 124,281 patients visited the kwaDukuza PHC clinic for a wide variety of primary health care reasons. Approximately 71% of those visits were made by patients over the age of fifteen. Budget planning for the clinic is the responsibility of the head nurse and is performed on a yearly basis. The annual budget for HIV/AIDS programs is estimated at US$ 716,000 for the year 2004. Detailed budget calculations are provided in the Appendix.

As of April 2005, six HIV/AIDS programs were offered at the clinic: voluntary counselling & testing (VCT), a prevention measure aimed at getting people to know their HIV status; antiretroviral therapy (ART) administered through a nearby hospital; condom distribution; short course antiretroviral treatment for the prevention of mother-to-child transmission (PMTCT); treatment of sexually transmitted infections (STIs), used to decrease the probability of HIV transmission; and a "wellness" program intended to prevent and treat opportunistic infections (OI) in people living with HIV/AIDS.

Annual data on the utilization of HIV/AIDS programs at the kwaDukuza PHC clinic for the year 2004 are summarized in Table 1. Data were obtained from clinic nurses and the information officer for the municipality. According to this data, 57% of those tested for HIV through VCT were positive and 40% of the women tested through the PMTCT program were positive. A total of 4741 patients attended the clinic for STI related reasons, and of those 68% received treatment. Condoms are distributed in packages of ten and are accessible in common areas of the clinic. In 2004, 4664 packages were taken. Assuming that at most one package is picked up by patients above the age of 15 who attended the kwaDukuza PHC clinic in 2004, then 5.24% of patients took a package of condoms.

**Table 1:** HIV/AIDS program utilization at the kwaDukuza PHC clinic (Y2004)

HIV/AIDS program	2004 annual estimate (Supplied by iLembe District Health office)	
VCT	Number of patients counseled	1,728
	Number of patients counselled & tested	1,587
	Number of patients tested HIV positive	897

ART	Number of patients with clinical AIDS	7,364
	Number of patients referred for ART	180
	Number of patients in ART program (administered outside clinic)	540

Condom	Condom packages picked up	4,664

Wellness (Annual estimate based on 6 months of data)	Number of patients seen	4,880
	CD4 cell count tests	724
	Patients with CD4 cell count <200	276

Treatment of STIs	Number of patients seen for STIs	4,741
	STIs treated (new episode)	3,203
	STI partner notification slips issued	4,288

PMTCT (Annual estimate based on 9 months of data)	Number of patients counselled	2,840
	Number of patients counselled & tested	2,540
	Number patients tested HIV positive	1,056
	Number of patients given nevirapine	732

National guidelines for the initiation of antiretroviral therapy include a CD4 cell count of less than 200 per cubic millimetre of blood. Of the patients seen by nurses or HIV/AIDS counsellors in the wellness program, 15% had their blood drawn for CD4 cell count testing. Of those tested, 38% had a CD4 count below 200 and 65% of those were referred for antiretroviral therapy. The public ART program in the municipality of kwaDukuza began in April 2004 and is administered by the main hospital located near the kwaDukuza PHC clinic. As of March 2005 there were 540 patients in kwaDukuza receiving ART through the public health system.

### Step 2: Influence diagram

**Figure 2:**
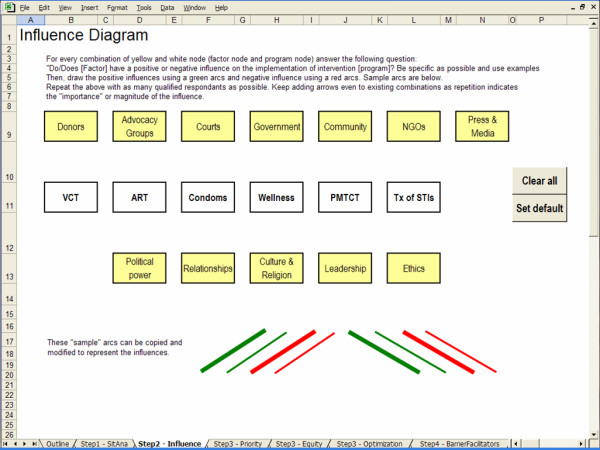
Initial blank screen of an influence diagram.

**Figure 3:**
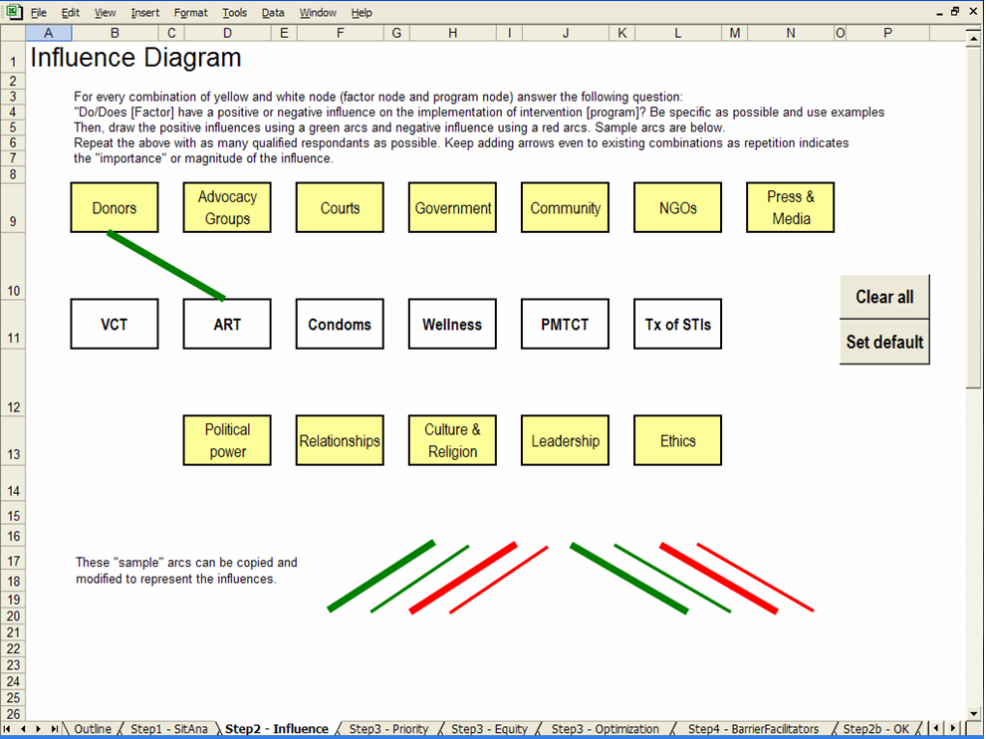
Positive influence arc.

**Figure 4:**
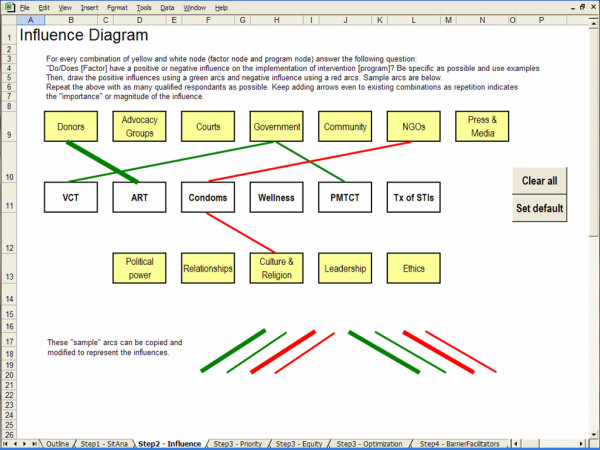
Adding arcs to create an influence diagram.

**Figure 5:**
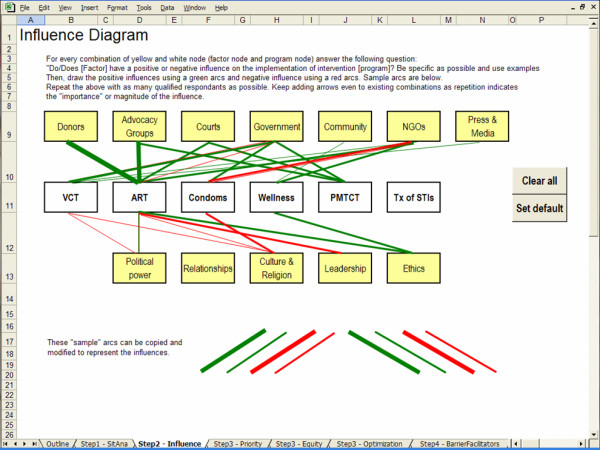
Finalized influence diagram.

The screen captures in Figures 2, 3, 4 and 5 illustrate the creation of an influence diagram. Figure 2 shows the initial screen containing only program nodes and factor nodes. For every combination of program and factor node, we determined whether there is a positive or a negative influence between the factor and the program. This assessment of the influencing factors is based on information from a case study of kwaDukuza [24]. For example, donor organizations are funding medical equipment, infrastructure improvements and temporary human resources to support the enrolment of patients in the ART program. Therefore, donors have a strong positive influence on the expansion of the ART program at the kwaDukuza PHC clinic. Accordingly, we draw a green arc from the Donors node to the ART node, as shown in Figure 3. The provincial and district levels of government have enforced PMTCT and VCT programs in all primary health care clinics so we draw green arcs from the Government node to both the PMTCT and VCT nodes, as in Figure 4. Cultural standards, social stigma and some faith-based organizations have a negative influence on condom uptake in kwaDukuza so we draw red arcs from the NGO and Culture & Religion nodes to the Condom node, as in Figure 4.

We proceed with an evaluation of all possible combinations of programs and factors, drawing arcs wherever applicable. We use thin and thick arcs to represent minor and major influences. The final influence diagram is illustrated in Figure 5.

### Step 3: Resource allocation

In this step, the user selects and applies one of three approaches to HIV/AIDS resource allocation. For the sake of demonstrating the use of S4HARA, we outline the application of each approach in the subsections below. Results of the three approaches are described with the help of S4HARA screen captures reflecting the completed allocation.

#### Priority setting

**Figure 6:**
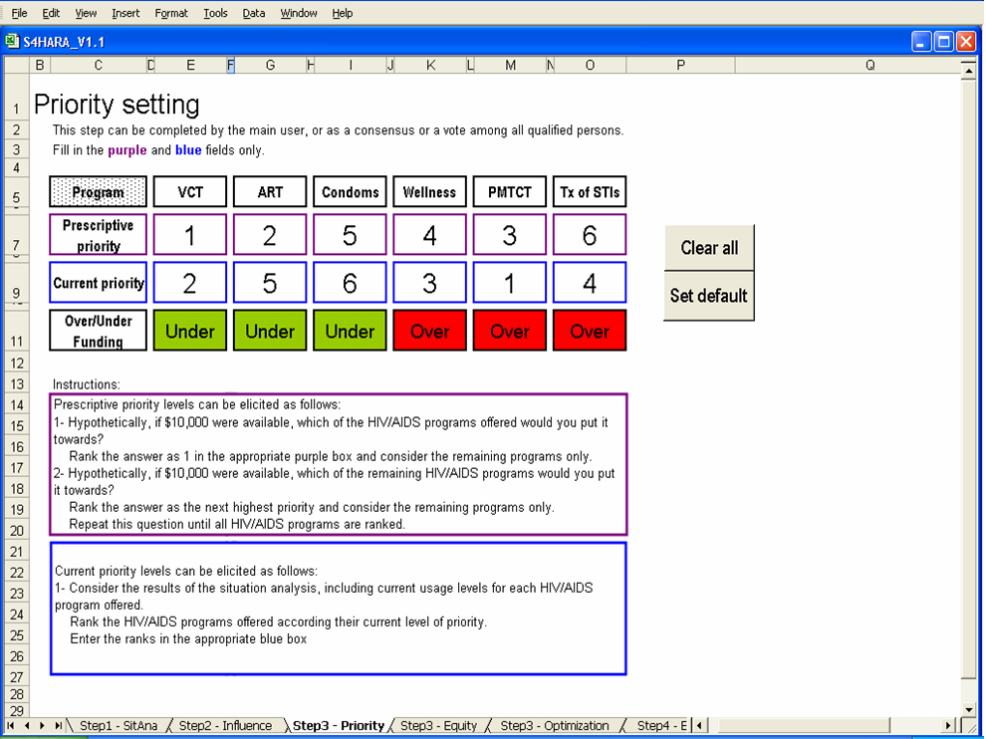
Priority setting as the approach used in Step 3.

Figure 6 is a screen capture of S4HARA showing the results of Step 3 when the priority setting approach is applied to the kwaDukuza PHC clinic. Data entry in this step is limited to the Prescriptive priority and Current priority fields. The background color of fields in the Difference row is highlighted in green when a program should be given more funding to meet the prescriptive priorities while it is highlighted in red when a program should be given less priority.

Prescriptive priority fields were assigned based on the elicitation method outlined earlier. Results of the situation analysis and knowledge of current processes at the kwaDukuza PHC clinic helped determine the Current priority fields.

According to the results highlighted, VCT and condom distribution should receive slightly more resources and the ART program should receive significantly more resources. These additional resources can be reallocated from the three other programs.

#### Equity

**Figure 7:**
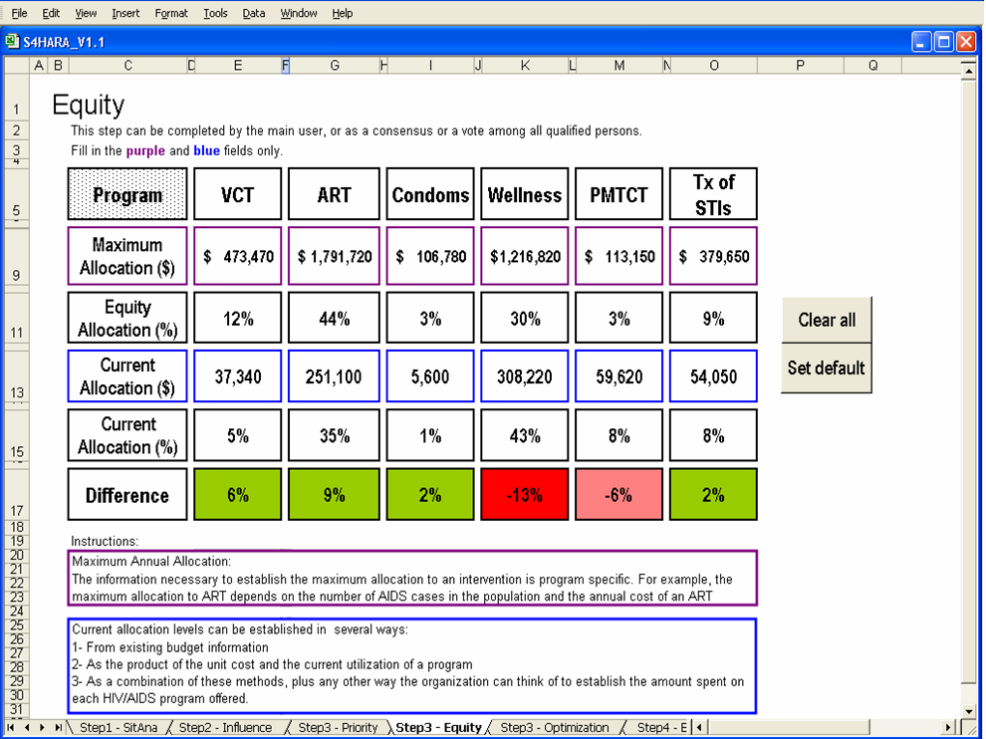
Equity as the approach used in Step 3.

Figure 7 shows the allocation of resources when the equity approach is applied to the kwaDukuza PHC clinic. The background color of fields in the difference row is highlighted in green when a program's share of the budget should be increased to meet the equity allocation while it is highlighted in red when a program's share should decrease.

The maximum allocation to each program assumes a time horizon of one year and is the product of the maximum number of people that could be reached by a program and the unit cost of the program. For a given HIV/AIDS program, the current allocation is the product of the program utilization and the unit cost of the program. Program utilization rates were summarized in Table 1. The unit cost of HIV/AIDS programs are derived from a recent study of the impact of scaling up HIV/AIDS intervention programs in low- and middle-income countries [41]. These data are displayed in the Appendix.

The results highlighted in the difference row indicate that VCT, ART, condom distribution and the treatment of STIs should receive a greater share of the available budget while the remaining two programs should receive less funding. Although these results may seem unfair to some population subgroups, they may represent improved distributive justice to others because the resources are allocated to the HIV/AIDS programs on the basis of equity.

#### Optimization

Data requirements for optimizing the allocation of resources to the HIV/AIDS programs include: cost-effectiveness ratios; minimum and maximum funding levels for each program; current allocation to the programs; and a total budget constraint. The kwaDukuza PHC clinic does not conduct economic evaluations so cost-effectiveness ratios for HIV/AIDS programs in similar settings were identified from the secondary sources [25,26,42,43]. We standardized the cost-effectiveness ratios by converting all costs to US dollars for the year 2004 and converting outcomes into DALYs as needed. We used these ratios to calculate an average cost per DALY saved. Results are summarized in Table 2.

**Table 2:** HIV/AIDS program cost per DALY saved as reported in the literature (US$ 2004)

HIV/AIDS cost-effectiveness studies					
HIV/AIDS program	Hogan (2005)	Creese (2002)	Badri (2006)	Marseille (2002)	Average cost per DALY saved

VCT	$89.97	$21.16	-	-	$55.56
PMTCT	$37.30	$7.72	-	-	$22.51
Treatment of STIs	$20.85	$13.49	-	-	$17.17
ART	-	-	$0^1^	$370.20	$185.10
Wellness	-	$6.48	-	-	$6.48
Condom distribution^2^	-	-	-	-	$4.60

^1 ^We used a value of zero because Badri et al. (2006) report that ART is cost-saving relative to a "no intervention" comparator (no-ART). According to this study, the yearly cost per person of a patient on ART is $1513 while is it $3520 for no-ART due to increased medical costs associated with the treatment of opportunistic infections. Further, the number of survival days is 1120 for a patient on ART and 510 days when a patient is not on ART. The resulting incremental cost-effectiveness ratio is negative indicating that ART is both less expensive and more effective than no-ART.

^2 ^The cost per DALY saved for the condom distribution program is calculated by translating an averted infection into DALYs gained.

**Figure 8:**
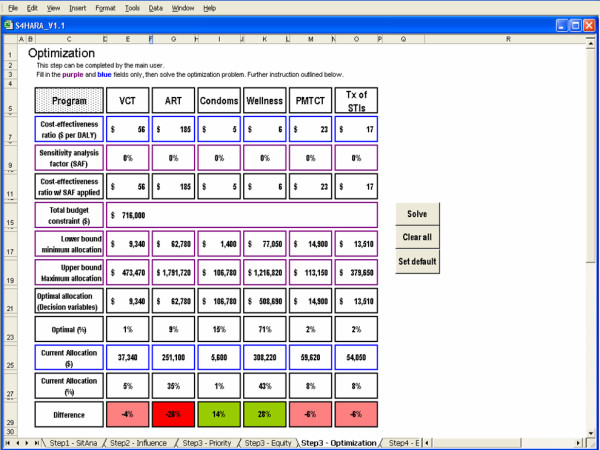
Optimization as the approach used in Step 3.

The optimal allocation of resources to the HIV/AIDS programs offered at the kwaDukuza PHC clinic is shown in Figure 8. The total budget constraint is set as the sum of the current allocation to the HIV/AIDS programs. Since we did not want to entirely eliminate the allocation to a program, we introduced a lower bound on the allocation to each program set at 25% of the current funding level. This lower bound is arbitrary and ultimately a decision of the user.

As well, we limited the allocation to each program using an upper bound set as the product of the unit cost and the maximum possible usage for each program. For example, the upper bound to the condom distribution program is set as the product of the cost per male condom distributed and the maximum number of condoms that can potentially be distributed by the clinic in a year. According to the unit cost data in Table 3, the median cost per male condom distributed in sub-Saharan Africa is $0.12. In 2004, approximately 88,985 visits were made by patients over the age of fifteen to the kwaDukuza PHC clinic. Assuming that the maximum number of condoms that can possibly be distributed is attained if every adult patient picks up a package of ten condoms during their visit, then 889,850 condoms is the maximum. Thus, the upper bound to the condom distribution program is 889,850 condoms × $0.12 per condom = $106,782.

**Table 3:** Unit costs of HIV/AIDS programs

**Median unit costs by HIV/AIDS program in sub-Saharan Africa** [41]		
Condom distribution	Cost per male condom distributed	$0.12
Treatment of STIs	Cost per STI treated in clinic	$11.40
VCT	Cost per person counselled and tested	$21.61
PMTCT	Cost per woman screened	$8.00
	Cost of ARV regimen per woman testing HIV+	$10.00
	Cost per woman of six months of infant formula	$69.60

**Average unit costs HIV/AIDS program in sub-Saharan Africa(2004 US$) **[41]		

ART	ARV first line (per patient yearly cost)	$449.00
	Labs for ARV monitoring (per patient yearly cost)	$16.00
Wellness	Prophylaxis of OIs (per patient yearly cost)	$71.00
	Treatment of OIs (per patient yearly cost)	$415.00

The background color of fields in the difference row is highlighted in green when a program's share in the budget should be increased to meet the optimal allocation. It is highlighted in pink when a program's share should decrease slightly, while it is highlighted in red when a program's share should decrease by more than 10% relative to the current allocation.

The optimal allocation suggests that condom distribution and the wellness program should receive a greater share of the available budget and the ART program should receive a significantly smaller share. Since the average cost per DALY saved for the condom distribution and wellness programs is less than that of the remaining programs, then allocating funds to condom distribution and wellness is a more efficient use of resources. For example, it is more cost-effective to gain a DALY by spending $4.60 on condom distribution rather than gain a DALY by spending $55.56 on VCT (see Table 2). The total number of DALYs gained according to the current allocation is 57,000 while the optimal allocation yields a total of 104,000 DALYs, representing an 83% increase in the number of DALYs gained.

**Figure 9:**
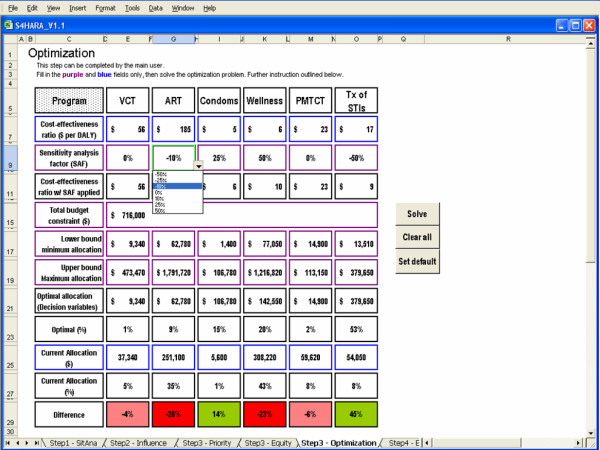
Sensitivity analysis on Optimization as the approach used in Step 3.

To demonstrate sensitivity analysis capabilities, we increased the cost-effectiveness ratios for condom distribution and wellness by 25% and 50%, respectively, and decreased the cost-effectiveness ratios for the ART program and the treatment of STIs by 10% and 50%, respectively. This scenario causes a reordering of the intervention ranking and optimal results suggest a greater allocation to the treatment of STIs and a reduction in the allocation to the wellness program. Results are shown in Figure 9.

### Step 4: Barriers and facilitators

**Figure 10:**
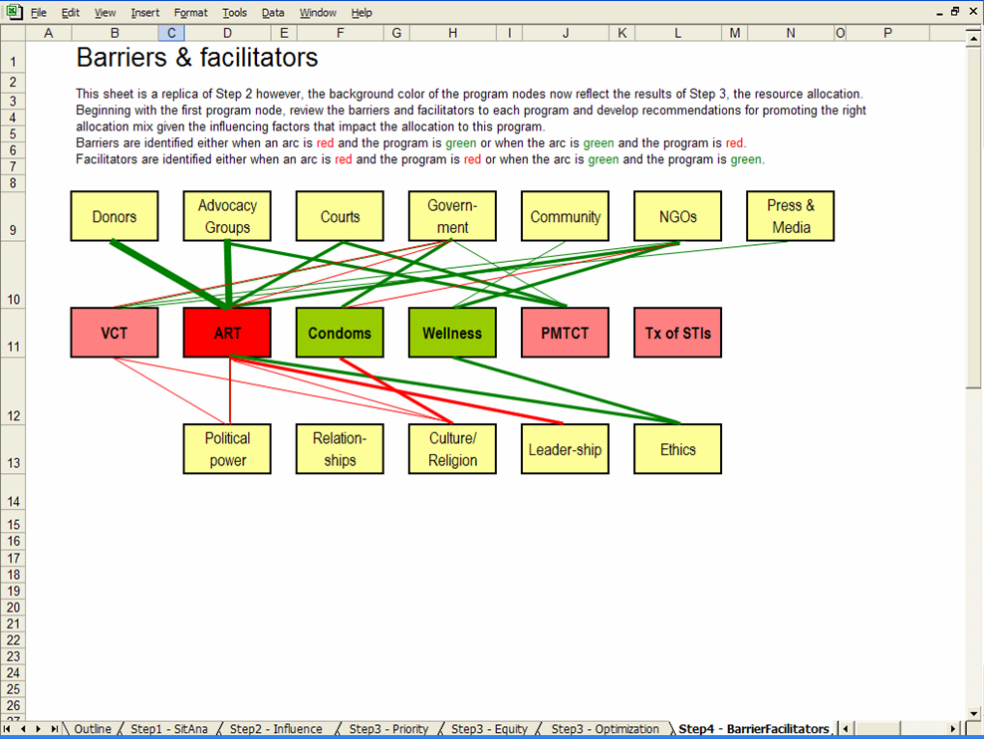
Barriers and facilitators.

The results of this step are described assuming that the previous step was completed using the optimization approach. The influence diagram shown in Figure 10 is a replica of the results of Step 2. However, the program nodes reflect the results of the optimal resource allocation (Step 3). To complete this step we review the influence diagram, identify the sources of disparity between the program nodes and the influencing factors and make recommendations to reduce these disparities. Our recommendations are as follows. We highlight three of the most important influencing factors, although others may exist.

First, donors and advocacy groups promote the ART program while the optimal resource allocation suggests that funding be reallocated from the ART program to condom distribution and wellness. Therefore, donors and advocacy groups act as barriers to the optimal solution. We recommend improved communication between the clinic and its donors that is assisted by the model's results. Using the model, the clinic can support the argument that donors may improve their return on investment by reallocating some funds to condom distribution and wellness. In addition, we recommend the clinic invite the advocacy groups to review and discuss the outcome of S4HARA. As shown in Figure 10, ethical considerations bear a positive influence on the ART program and in part, these considerations motivate advocacy groups to lobby for the ART program. As such, it is unlikely that advocacy groups would reduce their appeal for ARTs on the basis of an optimal allocation. However, given their clout and impact in the community, they may agree to advocate for condom use and access to the wellness program in addition to the ART program.

Second, NGOs and the local community have a positive influence on the wellness program and are a facilitator to the objective set by the optimal solution. In kwaDukuza, several NGOs including faith-based organizations and community-based organizations have formed a networking forum which meets on a regular basis to share experiences and successes. To take advantage of these facilitators, we recommend that a representative from the clinic attend a meeting of the networking forum to present the benefits of the wellness program and ask the organizations to refer HIV positive patients to the program.

Third, culture is an important negative influence on condom distribution. Currently, condoms are distributed by making them available in public areas of the clinic but embarrassment acts as a barrier and limits access to condoms. According to the situation analysis, at most 5% of adult attendees to the clinic took a package of condoms. We recommend that the clinic change the condom distribution policy from a "pull" system to a "push" system. That is, we suggest that health care workers at the clinic hand packages of condoms to all the adult clinic attendees rather than rely on them to pick them up.

## Discussion

We developed S4HARA, a system which differentiates itself from traditional resource allocation models in that it combines the results of a rational model to the non-quantifiable factors that affect allocation decisions. S4HARA's comprehensive solution is favourable to the formulation of recommendations for improving HIV/AIDS resource allocation. These recommendations are grounded in the cultural, social and political context, and thus are more likely to be adopted than recommendations derived from traditional approaches that ignore these non-quantifiable factors.

The kwaDukuza PHC clinic could improve the allocation of resources to HIV/AIDS programs and maximize population health by taking the following actions: meet with donors and advocacy groups to promote condom distribution and wellness programs, meet with the NGO networking forum of kwaDukuza to increase referrals to the wellness program, and change the condom distribution policy from a "pull" system to a "push" system. These recommendations are the main output of applying S4HARA to the kwaDukuza PHC clinic using optimization as the rational resource allocation model in Step 3.

Decision-making for HIV/AIDS resource allocation occurs at the several levels including that of the institution and of health systems. S4HARA is designed to support decision-making at the institutional level because at that level it is easier to identify and manage factors that act as barriers or facilitators to an improved resource allocation process. Further, resource allocation strategies at the lower level have a greater impact on overall health outcomes in a two-level resource allocation framework [12]. These results are similar to those obtained elsewhere [44].

Microsoft Excel version 2000 or above is the only software requirement for running S4HARA. However, this requirement may not be met in some low-income settings where there is limited access to computers. In this case, a printed workbook version of S4HARA could be developed. In this case, the resource allocation method chosen would likely be priority setting or equity as these approaches requires the least data.

S4HARA has several limitations. First, the recommendations drawn from Step 4 are susceptible to variability in the data, particularly the cost-effectiveness information as it drives the optimal allocation of resources. For example, if the cost of administering ART were to reduce dramatically, the intervention program would have greater priority in the optimal allocation of resources. Though sensitivity analysis helps assess the impact of data variations and the robustness of the recommendations. Second, the optimization model in Step 3 assumes that costs and outcomes are a linear function of amount allocated to a program. This assumption is restrictive given the non-linear behavior that epidemic growth curves reveal over time. However, the optimization method chosen in S4HARA is simple and when used over relatively short time horizons provides a piecewise linear approximation of the underlying nonlinear relationship. Also, we presented S4HARA using one possible definition of equity. However, S4HARA is a flexible spreadsheet-based model, so the user could redefine equity allocation according to different criteria as needed. Lastly, S4HARA does not yield an automated solution as to how HIV/AIDS resource allocation can be improved upon. Rather, S4HARA helps decision-makers structure the problem. Their knowledge and experience with the institution, the targeted population and with HIV/AIDS program funding is mandatory to envision and conceive of the recommendations intended to improve the allocation.

Future directions for this research includes field testing of S4HARA and the development of detailed user documentation. In addition, S4HARA can be enhanced to consider the allocation of resources to new HIV/AIDS programs that are not currently funded.

This work is novel in its application of both rational modeling and empirical evidence to create a pragmatic approach to HIV/AIDS resource allocation. S4HARA combines quantitative and qualitative methods, and aims to increase the influence of models on the development of health funding policies, an issue not unique to financing HIV/AIDS in endemic areas. Though S4HARA has been designed to focus on HIV/AIDS resource allocation in resource-poor settings, the system could easily be adapted to other diseases in other settings. Generalizing the overall approach suggested to other health care settings could yield important benefits for research in resource allocation methods. We view the proposed system as a first step aimed at bridging the gap between resource allocation theory and policy practice.

## Appendix

### Mathematical formulation of the optimization model

In the optimization model, we aim to maximize the cumulative number of DALYS gained by allocating funds to the intervention programs considered. Let *x*_*i *_be the amount invested in intervention *i*. Let *c*_*i *_be cost-effectiveness ratio of intervention *i *expressed in $ per DALY gained. The optimization model is written as follows:

(1) $$\begin{array}{cc} {\text{Max}}_{x_{i^{\prime }}} & \sum_{i}x_{i}/c_{i} \end{array}$$

(2) $$\begin{array}{cc} \text{subject to}: & \sum_{i}x_{i}\leq B \end{array}$$

(3) *x*_*i *_≤ *max*_*i *_∀*i*

(4) *x*_*i *_≥ *min*_*i *_∀*i*

The decision variables *x*_*i *_represent the amounts allocated to the intervention programs. B is the total budget constraint. The allocated amounts are constrained by a maximum investment, in (*3*), and by a minimum investment in (*4*) where it is assumed that *min*_*i *_≥ 0. This linear optimization model is equivalent to a knapsack problem and is easily solved [39].

### Estimating the HIV/AIDS program budget

To begin, we estimate the kwaDukuza PHC clinic HIV/AIDS program budget for the year 2004 using method "A". We then estimate the kwaDukuza PHC clinic HIV/AIDS program budget for the year 2004 using method "B" and compare the result to that of method "A".

#### Method "A"

Unit costs of HIV/AIDS programs are derived from a recent study of the impact of scaling up HIV/AIDS intervention programs in low- and middle-income countries [41]. They are reproduced in Table 3.

To estimate the kwaDukuza PHC clinic HIV/AIDS program budget for the year 2004, we sum the expenditures on the different HIV/AIDS programs offered at the clinic. Spending on an HIV/AIDS program is the product of the program utilization rate and the unit cost of the program. Annual data on HIV/AIDS program utilization at the kwaDukuza PHC clinic for the year 2004 were obtained from clinic nurses and the information officer for the municipality of kwaDukuza. Table 4 shows the annual spending by program and a total estimated HIV/AIDS program budget of $715,923 (2004 US$).

**Table 4:** 2004 Estimated annual HIV/AIDS program budget at the kwaDukuza PHC clinic

HIV/AIDS Program	Program utilization rate	2004 estimate^3^	Program unit costs [41]	Current annual spending
Condoms	Number of condoms distributed	46,640	$0.12	$5,597
Treatment of STIs	Number of patients seen for STIs	4,741	$11.40	$54,047
VCT	Number of patients counselled	1,728	$21.61	$37,342
PMTCT	Number of patients counselled	2,840	$20.99^4^	$59,617
ART	Number of patients on ART	540	$465.00	$251,100
Wellness	Number of distinct patients seen^5^	2,440	$126.32^6^	$308,220
	Total HIV/AIDS program spending			$715,923

^3 ^2004 data supplied by the iLembe District Health office

^4 ^Of the total number of patients counseled and tested for PMTCT, 29% received a regimen of Nevirapine, see Table 1. According to a clinic nurse in charge of the PMTCT program, the uptake of infant formula is 50%; the remaining women are advised to breastfeed exclusively for 6 months. Therefore, the unit cost for PMTCT is: (8.00 + (0.29*10.00) + (0.29*0.50*69.60)) = $20.99

^5 ^In 2004, there were a total of 124,281 visits to the kwaDukuza PHC but the population targeted by the clinic is estimated at approximately 61,000. Therefore each person in the target region visited the clinic an average of two times in the year 2004. Further, 4,880 patients were seen in the wellness program in 2004, see Table 1. The unit costs for wellness in Table 3 are estimated on a per patient basis; thus we refer to the number of distinct patients seen in the wellness program (4880 visits ÷ 2 visits per patient = 2440 distinct patients).

^6 ^The unit cost of treatment of OIs in Table 3 is estimated on a per patient lifetime basis. We assume the average number of life years remaining is 7.5 years. Therefore the yearly unit cost of the wellness program is: (71.00 + (415 ÷ 7.5)) = $126.32

#### Method "B"

In South Africa, there are four levels of government: national, provincial, district and municipal. The province of kwaZulu-Natal is comprised of ten districts including the iLembe District. The municipality of kwaDukuza is one of four local municipalities in the iLembe District.

In order to validate the total budget estimated using method "A", we use method "B" to estimate the HIV/AIDS budget and compare the outcomes. Method "B" consists of allocating part of the provincial HIV/AIDS budget to the kwaDukuza PHC clinic according to equity criteria.

We gather HIV/AIDS spending data for province of kwaZulu-Natal (KZN), then estimate the total provincial HIV/AIDS budget for the fiscal year 2004–2005 at $71 million, see Table 5.

**Table 5:** 2004/2005 HIV/AIDS budget for the province of kwaZulu-Natal (KZN)

	HIV/AIDS budget 2004–05 (×1000 2004 US$)
KZN Department of Health	$54,125 [47]
KZN Department of Education	$4,504 [48]
KZN Department Social welfare	$2,024 [47]
Foreign assistance (Global Fund)	$10,549 [47]
Total	$71,202

The population of kwaZulu-Natal is estimated at 9.5 million while the population of the district of iLembe is estimated at 577,000, or approximately 6% of the total [45,46]. If the HIV/AIDS budget for kwaZulu-Natal is allocated to the districts proportionally to their population, then district of iLembe's share of the budget would be 6% of $71 million or approximately $4.3 million.

There are 25 primary health care clinics in the district of iLembe, each delivering HIV/AIDS programs. In 2004, a total of 1,088,546 patients visited these clinics, and 11.4% of those visits were made at the kwaDukuza PHC clinic. If the HIV/AIDS budget for district of iLembe is allocated to the primary health care clinics in the district kwaDukuza PHC clinic proportionally according to the number of visits, then the kwaDukuza PHC clinic's share of the budget would be 11.4% of $4.3 million or approximately $494,000. However, this estimate excludes $251,100 in expenditures for the ART program because the program is managed through a nearby hospital (see Table 4). Therefore, we adjust the estimated kwaDukuza PHC clinic budget by adding the annual spending on the ART program (Table 4). The adjusted HIV/AIDS budget estimate for the kwaDukuza PHC clinic is $745,100.

The HIV/AIDS budget estimate for the kwaDukuza PHC clinic using method "A" is approximately $716,000 while it is $745,000 using method "B". We conclude that the initial method used is valid in estimating the 2004 annual HIV/AIDS budget for the kwaDukuza PHC clinic.

### Maximum allocation to a program

The maximum annual allocation to each of the HIV/AIDS programs is the product of the maximum number of people that can potentially be reached and the unit cost of the program. Unit costs are defined in Table 3. The maximum annual allocation to the programs offered at the kwaDukuza PHC clinic are defined in Table 6.

**Table 6:** Maximum annual allocation to HIV/AIDS programs

HIV/AIDS Program	Maximum Allocation	Data and assumptions (Data supplied by the iLembe District Health office)
Condoms	$106,782	In 2004, there were approximately 88,985 visits the kwaDukuza PHC clinic by people over the age of 15. Condoms are distributed in packages of ten.
Treatment of STIs	$379,650	STI prevalence rate is 38% [46]. We assume an average of two STI recurrences per patient per year.
VCT	$473,466	The 15 and older population targeted by the clinic is 43,820 and we assume that at best they are tested for HIV on a biennial basis.
PMTCT	$113,145	In 2004, 2840 women were counseled. We assume the conversion rate from counseling to test can be increased is 100%. We assume that all women who test positive (40%) are treated with Nevirapine and offered infant formula.
ART	$1,791,716	The 15 and older population targeted by the clinic is 43,820 and 40% of them are HIV positive (HIV+). The size of the 0–15 population targeted by the clinic is 17,380 and we assume that 10% of them are HIV+. We assume that 20% of those HIV+ have AIDS and qualifies for ART.
Wellness	$1,216,821	We assume that 50% of the HIV + population in kwaDukuza PHC catchment area can benefit from the wellness program.

## Authors' contributions

AL conducted the primary field research, developed the model and wrote the first draft of the manuscript. GSZ and MWC participated in the design and coordination of the model. All authors read and approved the final manuscript.
